# Working: Glimpses of the pandemic from this fine place so far from home

**DOI:** 10.1177/1473325020973325

**Published:** 2021-03

**Authors:** Miranda Mosier

**Affiliations:** School of Social Work, Portland State University, Portland, USA

**Keywords:** Social work education, social justice, story-telling, narrative, counter narratives, working class

## Abstract

This manuscript was written for a special issue on Reflections on a Pandemic. In it, I write as an emerging scholar from a working-class background. The pandemic has underscored the divergence between my working life as an academic, which is unintelligible to those I love, and their “essential” work, which increasingly renders them expendable. In this essay I struggle with the tensions that other working-class scholars have articulated before me: I am tentatively welcome in a place that asks, or even demands, that I become someone whose work is unrecognizable to my loved ones. Through the use of reflective inquiry and (counter) narratives, I am working to alter social work education, creating space for others from working-class backgrounds who might find themselves in this fine place so far from home.

“I have come to believe over and over again that what is most important to me must be spoken, made verbal and shared, even at the risk of having it bruised or misunderstood.”

(Lorde, 1977/2007: 40)

My life has only recently come to resemble that of a “stranger in paradise” (Ryan and Sackrey, 1996): an academic from a working-class background. The pandemic has underscored a fundamental tension in my life: the growing divide between my daily life and the lives of those that I love. But the truth is, even in this fine place so far from home (Barney Dews and Leste Law, 1995), I am always tied to my working-class identity. As I consider how the pandemic has uprooted social work education and research and how to move forward, I draw strength from ancestors, both familial and scholarly, and attempt here to respond to Lorde’s (1977/2007) invitation to break the silences, with glimpses of the pandemic, from a working-class perspective.

## Disruptions: Teaching and learning in times of precarity

When the stay-at-home orders come in mid-March, the collective (online) space of academia erupts in panic and anxiety. There are questions about how we will continue scholarship and instruction, especially those who have, overnight, added “teacher” and “child care provider” to their daily responsibilities. Educators attentive to student needs, especially those of us students seek out as a refuge (which is another way to say faculty that are members of an underrepresented group) note the enormity of the task before us: carrying on with instruction as if all our students have internet access, a living space that allows them to attend classes via Zoom, and aren’t essential workers themselves. I remember my own years as a graduate student, and it’s easy for me to image how the pandemic could sidetrack the already-slim educational possibilities for working people:

In one of early years in a doctoral program, I gathered with some of my cohort to welcome incoming PhD students. One raises their hand to ask how we live on the modest stipend, and heads start nodding around the room. I realize that I’m the only one who is used to living on so little. *Some students’ lives are always precarious.*

## Distractions: Who are you calling fragile?

The sense of dread in academia contrasts starkly with the reactions of my loved ones, whose lives remain largely unchanged, except for the masks they now carry in their cars as they drive to work at the nursing home, as landscapers, and grocery store clerks. Suddenly I’m the one – the family member who made herself odd by first getting a bachelor’s degree and then staying in school to earn a doctorate – who is non-essential. The pandemic and my shift into remote work presents another fundamental difference between the world I now inhabit and the world of my loved ones. In the early weeks of the pandemic, driven by my colleague’s fear, I keep a COVID-19 tracking website minimized in my browser. Scrolling through it becomes a morning ritual. I cling to the unspoken belief that if I check the local, national, and global numbers of new cases and deaths regularly, then maybe I can somehow keep my family members safe. I carry the same anxieties my colleagues do about teaching and scholarship and how long this will all last. I also harbor the perhaps irrational fear that while I’m cocooned in my small, overpriced apartment in the rapidly gentrifying North of our city, teaching into a screen of tiny black boxes, that I will come out of this pandemic alive and some of my loved ones will not:

Early in my doctoral studies, I was introduced to the practice of research and scholarship through many visiting scholars. One scholar presented work drawing from the Fragile Families and Child Wellbeing dataset. Recognizing my own family in the inclusion criteria, I raised my hand to ask how the dataset was named. I was rebuffed later for asking an irrelevant question, and realize all these years later it was a question the scholar was probably unable to answer. While I am still in awe of academics, and this life that still promises the “paradise” Ryan and Sackrey (1996) spoke of, I’m still incredulous that people would be seen as categorically fragile, based on family forms. *The people I come from are anything but fragile.*

## Discoveries: What are the words you do not yet have? (Lorde,1977/2007: 41)

Perhaps the pandemic and the divergence I see between my own life and the lives of those I love is one more invitation to speak up about the realities of being a working-class scholar and student. Even in a school of social work, the classed aspects of my life – patterns of family formation and decisions about work and education, which led me to complete a bachelor’s degree in my early 30 s as a mother of teens and become a grandmother in my early 40 s – are often treated like idiosyncratic quirks, rather than recognized as distinctive features of my working class background, with a cultural logic of its own as well as material consequences. bell hooks (2000) reminded us that class is “your behavior, your basic assumptions about life…your concept of a future, how you understand problems and solve them, how you think, feel, act” (Brown, 1974; as cited in hooks, 2000: 3–4). In this pandemic I see my loved ones conceiving of the future and solving problems in the ways that working people have always done: put your head down and keep working. When the pandemic hits, I do my best to do the same.

It is in these moments of working and growing isolation that I seek out those I think of as my new ancestors: other working-class academics. I find refuge in Laurel Johnson Black’s (1995) words, words I fear would be “bruised or misunderstood” (Lorde,1977/2007: 40) by colleagues and family alike, words that capture the tension that’s been at the center of my work, something I’ve timidly labeled “relational loss”:

“This is a story, one about love and fear. It’s about every child’s nightmare of losing her family and the ways in which the world I now tentatively try to live in tries to make that nightmare come true, to make it not a nightmare, but a dream, a goal.” (14).

Similarly, bell hooks (1994) argued that those of us in higher education from “undesirable” class backgrounds are encouraged to cut ties with our origins. Her refusal to do so, and invitation to other “non-materially privileged” students and scholars to believe in our ability to “alter the bourgeois settings [we] enter” (183) sustain me. Like Johnson Black (1995), my position in academia feels tentative, and the goal seems to be living a life that takes me further away from my family. And like hooks (1994), I believe in the possibility of altering the classroom, of practicing pedagogy that does not demean or dismiss teachers or learners from non-materially privileged backgrounds:

I get very little sleep in the days before my interview for a tenure track job. I toss and turn, my mind centered on one aspect of my job talk: the lines of a poem I’m considering including. I hear my childhood in them: “pine trees, pine trees, pine trees” (Alexie, 2014: 27). Like the author, I spent parts of my early childhood in a forest, without indoor plumbing. It was poverty. But it was also “epic and gorgeous” (Alexie, 2014: 27). If I delivered these lines well, I might demonstrate a truth about my background that wasn’t adequately represented in my education, where rural poverty was presented solely as a risk. But if I failed…the possibility seemed too silly to put into words: *How can a forty-year old still be afraid that telling a room full of social workers will result in being taken away from her family?*

The pandemic has laid bare the indignities and downright dangers of working for so many in the United States, particularly Black, indigenous, and people of color, and for working class people, whose “essential” nature is being revealed as “expendable.” In this moment, I want to know, where are the working-class people in social work education? How will our classrooms adapt to teaching during pandemic, when poverty, racism, and inequality are more determinative than ever of our life chances, if those of us with lived experiences of poverty remain silent? Can we speak honestly about the experiences of workers, and what it feels like to be social worked? I will never forget. Not even in this fine place, so far from home.
